# Retinal Ganglion Cell Senescence Links Diabetes to Retinal Neurodegeneration

**DOI:** 10.7759/cureus.96926

**Published:** 2025-11-15

**Authors:** Ayana Suzumura, Hideyuki Shimizu, Kazuhisa Yamada, Junya Ota, Seina Ito, Koji M Nishiguchi, Hiroki Kaneko

**Affiliations:** 1 Department of Ophthalmology, Nagoya University Graduate School of Medicine, Nagoya, JPN; 2 Department of Ophthalmology, Hamamatsu University School of Meidicine, Hamamatsu, JPN

**Keywords:** diabetic retinal neurodegeneration, diabetic retinopathy, p16 gene, retinal ganglion cell, senescence-associated secretory phenotype (sasp)

## Abstract

Background

Diabetic retinopathy (DR) is a leading cause of blindness worldwide and traditionally considered a microvascular complication. However, accumulating evidence indicates that retinal neurodegeneration is also crucial in DR pathogenesis. Retinal ganglion cells (RGCs), the output neurons of the retina, are particularly vulnerable to diabetic stress. Cellular senescence has been implicated in diabetes-related tissue damage, but its contribution to RGC degeneration remains unclear. We hypothesized that diabetes contributes to retinal neurodegeneration by inducing senescence in RGCs.

Methods

In streptozotocin (STZ)-induced diabetic mice, retinal function was assessed via full-field electroretinography (ERG), and molecular changes were evaluated in senescence markers. The expression of p16^INK4a^ and monocyte chemotactic protein-1 (MCP-1) in retinal tissue was evaluated by enzyme-linked immunosorbent assay (ELISA) and quantitative real-time polymerase chain reaction (qRT-PCR), and the localization of p16^INK4a^ was confirmed by immunostaining. To explore the direct effects of senescence, primary RGCs isolated from rat retina were exposed to oxidative stress or treated with the CDK4/6 inhibitor palbociclib. The isolated RGCs were analyzed via senescence-associated β-galactosidase (SA-β-gal) staining and live-cell neurite imaging.

Results

The STZ-induced diabetic mice exhibited significant hyperglycemia without weight loss. ERG revealed markedly reduced amplitudes of the a-wave, b-wave, and oscillatory potentials, indicating impaired retinal neural function. Molecular analyses revealed significant upregulation of MCP-1 and p16^INK4a^ at mRNA and protein levels. Immunostaining demonstrated p16^INK4a^ co-expression in a subset of NeuN-positive cells within the ganglion cell layer, suggesting RGC senescence. Palbociclib-induced senescence (confirmed by SA-β-gal positivity) in vitroresulted in progressive neurite shortening in RGCs. Similarly, oxidative stress induced by antioxidant-free culture conditions caused neurite degeneration, highlighting the dual contributions of oxidative stress and senescence to RGC injury.

Conclusions

Cellular senescence was identified as a critical mechanism underlying RGC dysfunction in diabetes. Diabetes was found to induce retinal senescence and senescence-associated secretory phenotype activation, with RGCs exhibiting senescence-associated changes. Moreover, oxidative stress and pharmacologically induced senescence directly impaired RGC morphology and function in vitro. These results expanded our understanding of DR from a solely vascular disorder to a neurodegenerative disease, providing mechanistic insights into the role of senescence in retinal aging and neuronal susceptibility in diabetes.

## Introduction

Diabetic retinopathy (DR) is a leading cause of blindness worldwide. The pathophysiology of DR is traditionally attributed to retinal microvascular damage, and its severity is clinically defined by the extent of microvascular abnormalities observed on fundoscopy. However, recent evidence suggests a close relationship between neurodegeneration and microvascular dysfunction from the early stages of DR, collectively termed “neurovascular unit impairment” [1]. The American Diabetes Association defines DR as “a highly tissue-specific neurovascular complication” [2]. Notably, the relationship between neurodegeneration and vascular abnormalities in DR is bidirectional, with neurodegeneration potentially preceding detectable microvascular changes [3]. Thus, early neuroprotective strategies can provide therapeutic benefits before the development of irreversible vascular complications.

Retinal ganglion cells (RGCs), which serve as the output neurons of the retina, are particularly vulnerable under diabetic conditions. Previous optical coherence tomography (OCT) analyses have demonstrated progressive RGC injury that manifests as thinning of the nerve fiber layer (NFL) in patients with diabetes, even among those with no or mild DR. This finding indicated that this neurodegeneration more closely correlates with the duration of diabetes rather than HbA1c levels [4]. Additionally, a meta-analysis of 47 studies conducted across 16 countries and involving approximately three million participants also reported a positive correlation between the duration of diabetes and the risk of glaucoma [5], drawing considerable attention to the effect of diabetes on RGC pathology.

Cellular senescence is an emerging mechanism implicated in diabetes-induced tissue damage. The core pathogenic processes of DR, such as mitochondrial dysfunction and oxidative stress, are known to induce premature senescence in retinal endothelial cells [6]. These senescent cells release inflammatory cytokines and chemokines, known as the senescence-associated secretory phenotype (SASP), which, in turn, promotes paracrine senescence and pathological neovascularization [7]. Among them, the chemokine monocyte chemotactic protein-1 (MCP-1) is a key SASP component known for recruiting immune cells and enhancing inflammation [8].

Therefore, this study aimed to determine whether diabetes induces cellular senescence in retinal neurons, particularly RGCs, leading to early neurofunctional impairment and identifying neuronal senescence as a potential therapeutic target.

## Materials and methods

Animal diabetic model

Eight-week-old male C57BL/6J mice (CLEA, Tokyo, Japan) were randomly assigned to diabetic or nondiabetic (control) groups. Diabetes was induced via intraperitoneal STZ (Sigma-Aldrich, MO) injection, wherein STZ was dissolved in 0.05 M sodium citrate buffer (pH 4.5) and injected at a dose of 150 mg/kg. The control group received an intraperitoneal injection of vehicle (citrate buffer control). Tail vein blood glucose was measured one week after the injection. Diabetes mellitus was confirmed based on a fasting blood glucose level of more than 250 mg/dL, as described previously [9]. The mice were euthanized by carbon dioxide inhalation after 12 weeks, and their retinal choroidal tissue was collected together for molecular analyses. All analyses adhered to the ARVO Statement for the Use of Animals in Ophthalmic and Vision Research and were conducted in accordance with the experimental protocol approved by the Nagoya University Animal Care Committee. In accordance with the 3R (Reduction) principle, we used the minimum number of animals needed to achieve statistically reliable conclusions. The large effect size observed in the preliminary experiments allowed us to detect statistically significant differences with a small sample size.

Electroretinography (ERG) measurement

We conducted ERG measurements as described previously [10]. The mice were dark-adapted for at least 12 hours and prepared under dim red illumination. After anesthesia with intraperitoneal ketamine and medetomidine, the pupils of the mice were dilated with topical 0.5% tropicamide/0.5% phenylephrine (Mydrin-P; Santen, Osaka, Japan), and the animals were placed on a heating pad. ERG measurements were recorded using a gold wire loop electrode placed on the cornea and a gold wire reference electrode on the sclera. Responses were amplified and subjected to bandpass filtering between 0.3 and 1,000 Hz. A computer-assisted signal-averaging system (PowerLab; AD Instruments, Castle Hill, Australia) was used to calculate the average ERG responses. For stimulation, a Ganzfeld bowl (Model GS2000; LACE Electronica sel via Marmiccilo, Pisa, Italy) with a xenon source was employed. The amplitudes of the a and b waves, the b/a ratio, and oscillatory potentials (OPs) were measured.

Protein and RNA isolation

For protein collection, the mouse retina was lysed in a RIPA buffer (Sigma-Aldrich, St. Louis, MO) supplemented with a protease inhibitor cocktail (Roche Diagnostics, Indianapolis, IN). The lysate was centrifuged at 15,000 rpm for 15 minutes at 4°C, and the supernatant was collected. Protein concentrations were determined using a bicinchoninic acid (BCA) protein assay kit (Takara Bio Inc., Otsu, Japan), using bovine serum albumin as a standard. Total RNA was extracted using the RNeasy Mini Kit (Qiagen, Hilden, Germany) as per the manufacturer’s instructions for subsequent quantitative real-time polymerase chain reaction (qRT-PCR). RNA concentration and quality were assessed using the NanoDrop ND-1000 spectrophotometer (NanoDrop Technologies, Rockland, DE).

Enzyme-linked immunosorbent assay and qRT-PCR

Mouse MCP-1 and p16^INK4a^ protein levels were quantified via respective ELISA kits (Abcam, ab100722 for MCP-1; ab230131 for p16^INK4a^) as per the manufacturer’s protocols. Retinal tissues were lysed to obtain protein extracts, and ELISA assays were conducted at room temperature until the final wash step was completed. The ELISA results were normalized per total protein concentration, and each sample was analyzed in duplicate. The ReverTra Ace® qPCR RT Master Mix with gDNA Remover (Toyobo #FSQ-301) was used for cDNA synthesis from RNA. In accordance with the manufacturer's protocol, the maximum recommended amount of 0.5µg of RNA template was used per reaction. The expression levels of MCP-1 and Cdkn2a (the gene that encodes p16^INK4a^ and p14ARF) were analyzed using TaqMan qPCR assays. The reaction mixtures (20 μL) contained TaqMan™ Fast Advanced Master Mix (Thermo Fisher Scientific, Applied Biosystems, Foster City, CA, #4444557), TaqMan Gene Expression Assay (Applied Biosystems, Foster City, CA), nuclease-free water, and template cDNA. The following TaqMan probes were used: mouse Cdkn2a(Mm00494449_m1), mouse MCP-1 (Mm00441242_m1), and eukaryotic 18s rRNA (Mm03928990_g1) (all from Applied Biosystems). qPCR amplification was performed on ausing the QuantStudio 5 Real-Time PCR System (Thermo Fisher Scientific, Applied Biosystems) with the following thermal cycling parameters: initial denaturation at 95°C for 20 seconds, followed by 55 cycles of 95°C for one second and 60°C for 20 s. Relative mRNA expression levels were calculated and normalized to the levels of the endogenous control gene 18s rRNA. The relative expressions of the target genes were determined by the 2^-ΔΔCt^ method.

Immunocytochemistry

The mice were euthanized, and their eyes were immediately enucleated. The eye cups were fixed in 4% paraformaldehyde and cryoprotected in 30% sucrose. Then, the eye cups were embedded in an optimal cutting temperature compound (Tissue-Tek OCT; Sakura Finetek, Torrance, CA), frozen, and cryosectioned into 10-μm sections. The sections were incubated with rabbit antibody against mouse NeuN (1:300 dilution; Abcam) and rat antibody against mouse p16 (kindly gifted from Dr. Giovanna Roncador), followed by appropriate species-specific Alexa Fluor 488 and 594 conjugates (1:1000 dilution; Thermo Fisher Scientific) and diamino-2-phenyl-indol (DAPI; 1:5000 dilution; Molecular Probes Inc., Eugene, OR). Finally, the sections were mounted using Fluorescence Mounting Medium (Dako, Carpinteria, CA) and analyzed using a fluorescence microscope (BZ-9000; Keyence, Osaka, Japan).

Purification of rat retinal ganglion cells

RGCs were prepared from Wistar rats (CLEA, Tokyo, Japan) five to six days postnatally using a two-step immunopanning method, as described previously [11,12]. The rats were euthanized via CO₂ inhalation, and the eyes were enucleated under sterile conditions. Retinal tissue was dissected and enzymatically dissociated into a single-cell suspension using a papain-based dissociation kit (Worthington Biochemical, Lakewood, NJ). The cell suspension was incubated with an anti-macrophage antibody (MAB1407P; Millipore, Bedford, MA) to remove the macrophages. Then, the non-adherent cells were incubated with an anti-Thy1.1 antibody (MAB1406; Millipore), and cells that bound to this antibody were identified as RGCs. The identified RGCs were cultured in Gibco Neurobasal Medium (Thermo Fisher Scientific, Waltham, MA), supplemented with 2% B27 (Thermo Fisher Scientific), 40 ng/mL brain-derived neurotrophic factor (Sigma-Aldrich, St. Louis, MO), 40 ng/mL rat ciliary neurotrophic factor (Sigma-Aldrich), 1 mM L-glutamine (Sigma-Aldrich), 10 mM forskolin (FUJIFILM, Santa Ana, CA), and antibiotics (100 U/mL penicillin and 100 μg/mL streptomycin; FUJIFILM). The purified RGCs were seeded at a density of 5.5 × 105 cells per well in 24-well plates and an 8-well Slide & Chamber (WATSON Bio Lab, Hyogo, Japan) coated with 50 μg/mL poly-L-lysine (Sigma-Aldrich) and 1 μg/mL laminin (Thermo Fisher Scientific). The purified RGCs were maintained in a CO₂ incubator, with medium changes performed every three days. After six days of culture, the RGCs were used for subsequent analyses. To simulate DR-related oxidative stress, RGCs were cultured without the antioxidant B27; the control group was cultured with 2% B27 added.

Induction of cellular senescence and evaluation of neurite length

To induce cellular senescence, purified rat RGCs were treated with 2 µM palbociclib as described previously [13]. Changes in the neurite length of RGCs were assessed for 12 h after palbociclib treatment using the Incucyte SX5 real-time imaging system (SARTORIUS, Germany). After imaging, neurite length was determined via the Incucyte Neurotrack Software (Sartorius, Göttingen, Germany) [9].

Senescence-associated β-galactosidase (SA-β-gal) staining

Five hours after administering 2 µM palbociclib or DMSO as a control, the purified rat RGCs were stained for senescence-associated β-galactosidase (SA-β-gal) at pH 6.0 using a commercial staining kit (Cell Signaling Technology, Danvers, MA, #9860) as per the manufacturer’s instructions. Briefly, 150 μL of staining reagent was applied to the well of each slide chamber, which was then covered with a coverslip and sealed with rubber cement to prevent evaporation. The slides were incubated in a sealed humidified chamber maintained at 37°C in a dry incubator (without CO₂) for 48 hours. Imaging was performed using an APEXVIEW APX100 Benchtop Fluorescence Microscope (Evident, Tokyo, Japan) at ×40 magnification.

Statistical analysis

The data were expressed as mean ± standard deviation (mean ± SD) and analyzed using the GraphPad Prism statistical software version 10.4.1 (GraphPad Software, Inc., San Diego, CA). The Mann-Whitney U test was used for comparisons between two groups, with p < 0.05 considered statistically significant. In part of the preparation of this paper, the author utilized ChatGPT (GPT-4o; OpenAI, San Francisco, CA) and Gemini (Gemini 2.5 Flash; Google, Mountain View, CA) to enhance the readability of the English text.

## Results

Diabetes decreased retinal function

**Figure 1:**
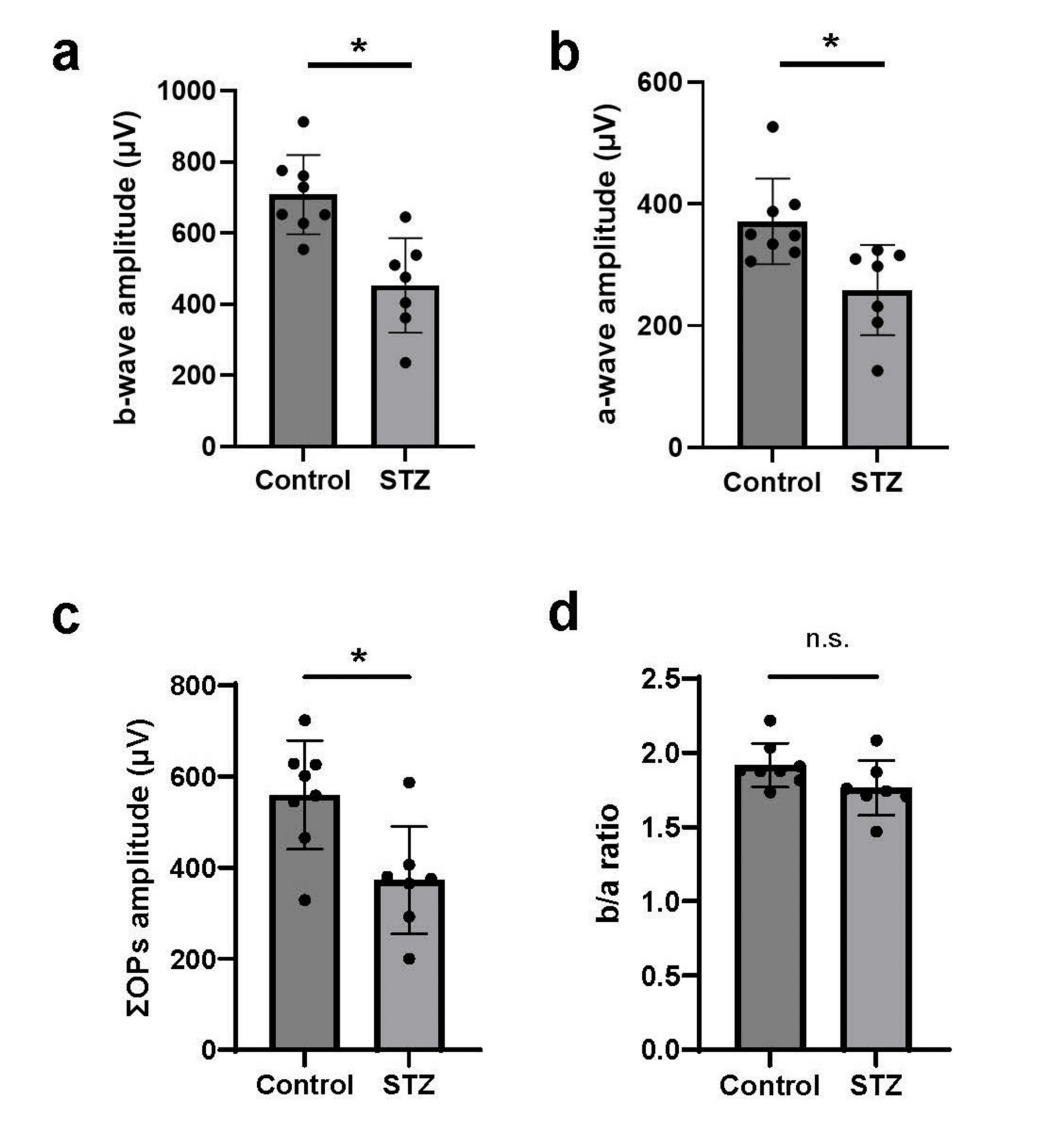
Retinal neuronal function deteriorates in streptozotocin (STZ)-induced diabetes. a-c: Full-field electroretinography (ERG) revealed a significant deterioration in the b-wave, a-wave, and OPs in STZ mice compared to the control. d: The b/a ratio was comparable between the two groups. Data represent mean ± SD. *p < 0.05. n.s., not significant

STZ-induced diabetic mice exhibited significant hyperglycemia but with no apparent reduction in body weight (Table 1). Full-field ERG was used to assess the effects of diabetes on retinal function. We observed that STZ mice exhibited significantly decreased amplitudes of the b-wave, a-wave, and OPs compared to control mice, indicating decreased neuroretinal function in DR (Figures 1a-1c). The b/a ratio was comparable between the two groups (Figure 1d).

**Table 1:** Changes in the body weight and blood glucose levels in the diabetic mouse model. Values represent mean ± SD. *p-value was <0.05 compared with the control. STZ, streptozotocin

	Control	STZ
STZ administration	-	+
Number of samples	8	7
Body weight before study (g)	23 ± 0.62	24 ± 0.75
Body weight after study (g)	26 ± 0.49	24 ± 1.8
Body glucose (mg/dL)	130 ± 16	481 ± 97.1*

Diabetes upregulated SASP and senescence markers in the retina

**Figure 2:**
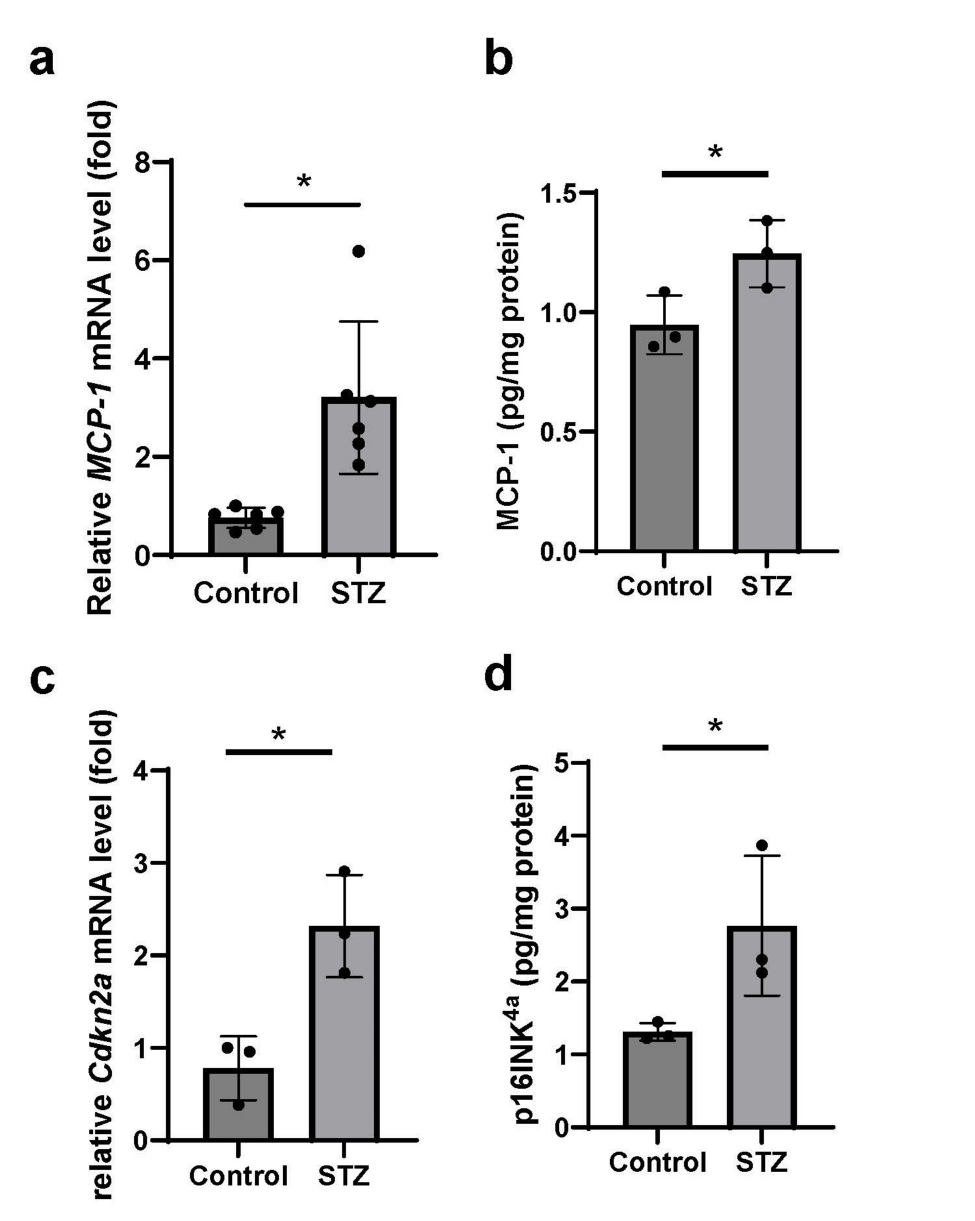
Evaluation of monocyte chemotactic protein-1 (MCP-1) and p16INK4a expressions in mice retinal choroidal tissue. a, b: The relative MCP-1 mRNA and protein levels were significantly higher in streptozotocin (STZ) mice than in control mice. c, d: The relative Cdkn2a mRNA and p16^INK4a^ protein levels were significantly higher in STZ mice than in control mice. Data represent mean ± SD. *p < 0.05.

Cellular senescence was evaluated as a potential mechanism underlying the retinal dysfunction in STZ-induced diabetic mice. Evaluation of MCP-1, a known component of SASP, revealed a significant upregulation of MCP-1 mRNA and protein in the retinas of STZ-induced diabetic mice (Figures 2a-2b). Similarly, the mRNA and protein levels of p16^INK4a^, a known marker of cellular senescence, were significantly upregulated in the retinas of STZ mice (Figures 2c-2d).

Cellular senescence in RGCs of STZ-induced diabetic mice

**Figure 3:**
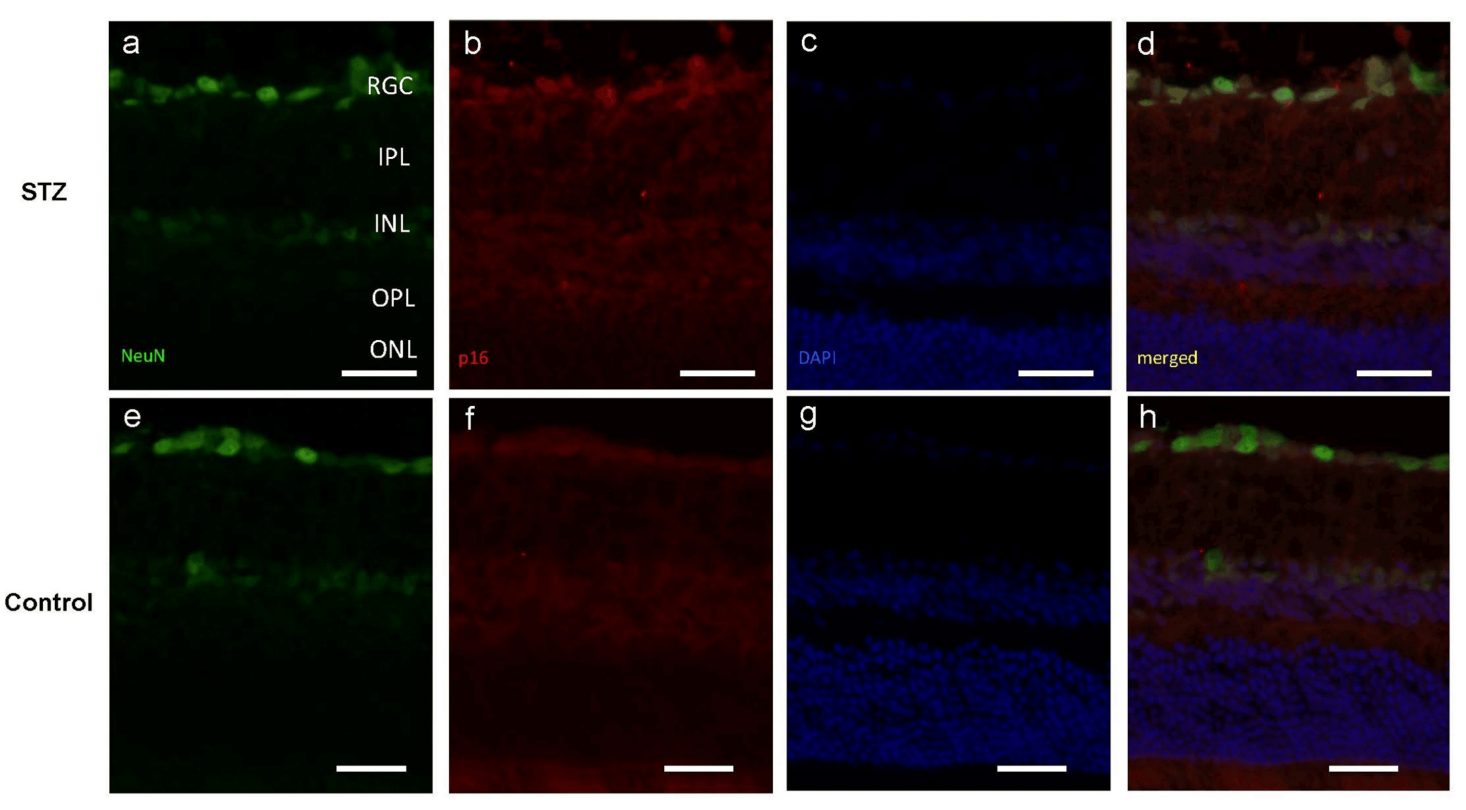
Co-localization of p16 and NeuN immunoreactivity in the mouse retina. a-d: Representative immunostaining of streptozotocin (STZ) mice retina. NeuN-positive cells were located in the outer ganglion cell layer, and partially co-localized with p16-positive cells. e-h: Representative immunostaining of control mice retina. NeuN-positive cells were located in the outer ganglion cell layer, and colocalization with p16-positive cells was not evident. Scale bars = 50 µm.

After confirming cellular senescence in the retinas of the diabetic model mice, we sought to identify the specific cell types undergoing senescence. In diabetic mice 12 weeks after STZ administration, immunostaining of retinal sections was performed using an anti-NeuN antibody, which predominantly labels RGCs in the ganglion cell layer (GCL), and an anti-p16^INK4a^ antibody. Our results revealed that a subset of NeuN-positive cells was also positive for p16^INK4a^ (Figure 3), suggesting that a portion of RGCs might undergo cellular senescence under diabetic conditions.

Changes in the neurite length of RGCs under cellular senescence and oxidative stress

**Figure 4:**
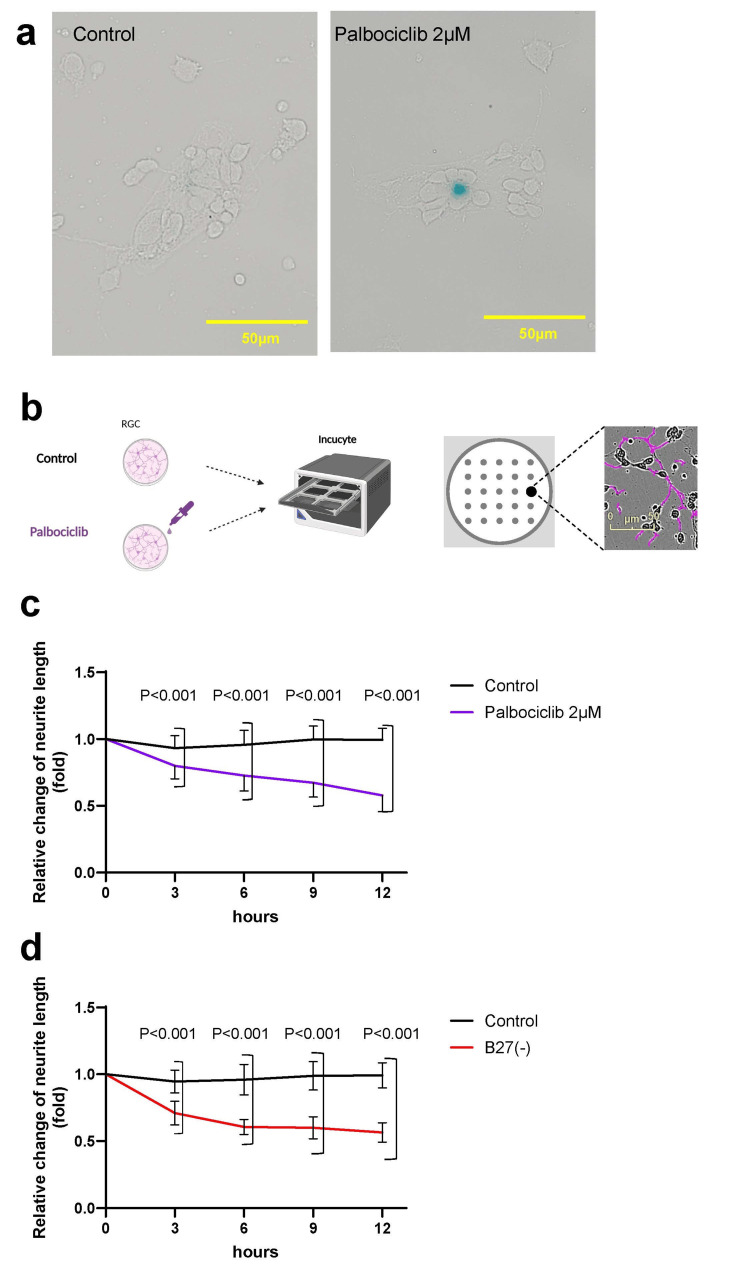
Neurite length of isolated retinal ganglion cells (RGCs) relatively decreased by palbociclib administration and oxidative stress. a: As confirmed by SA-β-gal staining, senescence was induced in RGCs by 2 µM palbociclib, a CDK4/6 inhibitor. Scale bars = 50 µm. b: RGCs were incubated in Incucyte for 12 hours, and changes in neurite length were assessed across 25 timepoints after palbociclib treatment. Neurite length was automatically determined via the Incucyte Neurotrack Software. c: RGCs demonstrated a significant decrease in neurite length after palbociclib administration in a time-dependent manner. d: RGCs demonstrated significantly decreased neurite length under oxidative stress.

In vitro experiments using RGCs isolated from rat retinas were performed to investigate the morphological changes in RGCs associated with cellular senescence. Senescence was induced by treating the cells with palbociclib, a CDK4/6 inhibitor, and confirmed via SA-β-gal staining. This approach is the most widely used assay for identifying senescent cells, as it helps detect increased lysosomal β-galactosidase activity characteristic of senescence [6]. Thus, SA-β-gal-positive senescent cells were observed in the group exposed to 2 µM palbociclib (Figure 4a). Thus, palbociclib at this concentration was used to induce cellular senescence in the subsequent analyses.

Changes in the neurite length of RGCs were evaluated via the Incucyte live-cell imaging system, which has an integrated analysis software that automatically detects neurites, enabling objective quantification (Figure 4b). Neurite length was assessed every three hours from the time of palbociclib administration. At all timepoints, the palbociclib-treated group exhibited significantly shorter neurites compared to the control group (Figure 4c).

To simulate DR-associated oxidative stress, RGCs were cultured in the absence of the antioxidant supplement B27. Neurite shortening was observed under this condition, indicating that oxidative stress also contributes to RGC degeneration (Figure 4d).

## Discussion

Recent evidence has established that DR is not merely a microvascular complication but also a neurodegenerative disease, and RGC impairment is known to occur even before vascular alterations become clinically evident [14]. Growing evidence has also highlighted the relationship between diabetes and glaucoma, a disease wherein RGC degeneration is the primary pathology. Specifically, diabetes has been identified as a risk factor for glaucoma independent of intraocular pressure [5,15]. Recent studies have revealed that cellular senescence plays a pivotal role in the pathogenesis of lifestyle-related diseases, including diabetes [16]. Building on these findings, the present study investigated cellular senescence as a potential mechanism linking diabetes to RGC degeneration. We first confirmed the impaired retinal function in STZ-induced diabetic mice. Our subsequent analyses revealed that senescence markers and SASP expression in the retina were significantly upregulated in the STZ mice compared to control mice. Furthermore, STZ-induced diabetic mice exhibited co-localization of the senescence marker p16^INK4a^ and NeuN, which predominantly reflects RGCs within the GCL. Lastly, the neurite length of isolated RGCs progressively decreased over time following palbociclib-induced cellular senescence, which, to our knowledge, was observed for the first time in this study.

Twelve weeks after STZ administration, the diabetic mice exhibited no significant differences in body weight or the ERG b/a ratio. However, the amplitudes of the a-wave, b-wave, and OPs were significantly reduced. The a-wave, b-wave, and OPs predominantly originate from photoreceptors, ON-bipolar and Müller cells, and amacrine cells, respectively. These findings indicated a widespread decline in retinal neural responses under diabetic conditions, extending from the outer to the inner layers [17]. However, the comparable b/a ratio between the diabetic and nondiabetic mice indicated that the outer and inner retinal dysfunction might progress relatively in parallel.

Furthermore, retinal cellular senescence was investigated at the molecular level. The mRNA and protein levels of MCP-1, an inflammatory chemokine, were significantly upregulated in diabetic mice, suggesting SASP activation in their retina. Similarly, the diabetic mice exhibited a significant upregulation of p16^INK4a^, a key marker of cellular senescence, thereby serving as molecular evidence of the progression of cellular senescence in diabetic retina. Based on these observations, we sought to identify the specific cell types undergoing senescence in the retina using double immunostaining for NeuN (a marker of mature central neurons, including RGCs) and p16^INK4a^. NeuN-positive cells in the GCL are generally interpreted as RGCs, although a small subset might represent displaced amacrine cells. Herein, NeuN immunoreactivity in the GCL was used as an indicator of RGCs. Our results revealed that a subset of NeuN-positive cells in the GCL co-expressed p16^INK4a^. Similarly, previous studies have reported p16^INK4a^ expression in the GCL of the retinal sections from patients with proliferative DR [18]. These findings suggested that a subset of RGCs undergoes senescence in diabetes, which aligned with previous clinical studies that have described the vulnerability of RGCs in diabetic retina [2]. Moreover, since senescent cells are known to contribute to retinal inflammation via SASP [19], our findings highlight the possibility that cellular senescence contributes to RGC damage caused by diabetes.

In vitro analyses using primary cultured RGCs revealed significant neurite shortening under oxidative stress induced by an antioxidant-free culture medium as well as palbociclib-induced senescence, evidenced by SA-β-Gal-positive cells. Therefore, oxidative stress, a major pathogenic factor in diabetes, and cellular senescence contribute to RGC damage, resulting in morphological alterations. Notably, this study demonstrated palbociclib-induced senescence in isolated RGCs. Collectively, these findings suggested that oxidative stress and cellular senescence synergistically impair the structure and function of RGCs, providing novel insight into the potential mechanisms that bridge hyperglycemia-induced stress and neurodegeneration.

Previous studies have demonstrated co-localization of the vascular endothelial marker isolectin B4 and p16^INK4a^ in retinal sections from STZ-induced diabetic rats [20]. Senescence has also been observed in vascular-associated cells (e.g., pericytes and endothelial cells) in an oxygen-induced retinopathy mouse model, which mimics DR [18]. Building on these pathological insights, senolytic agents targeting senescent cells are currently undergoing clinical investigation for their use in diabetic macular edema, a major ocular complication of diabetes [21]. Thus, interventions targeting cellular senescence, including senolytic therapies, have the potential to protect the retinal vasculature and exert neuroprotective effects to prevent or delay RGC impairment.

This study had several limitations. First, retinal function was assessed via ERG; however, this approach might not have fully captured RGC-specific functional changes. Although retinal function was evaluated using flash ERG, we did not extract the photopic negative response (PhNR), a slow negative component derived from RGCs that follows the flash ERG b-wave and serves as an indicator of RGC and axonal function [22]. Thus, subtle RGC dysfunction might have gone undetected in the ERG waveform. Aside from the PhNR, pattern ERG (pERG) has also been proposed as a more sensitive method for assessing RGC function [23]. Thus, PhNR and pERG are important for assessing RGC dysfunction, and our methodological approach may have overlooked RGC-specific functional impairment. Second, the experimental period was limited to 12 weeks after STZ administration, precluding the assessment of long-term changes in retinal neurodegeneration during the progression of diabetes. The observation window of this study only captured alterations that correspond to the early stages of DR, potentially overlooking any late-onset pathological or functional abnormalities. In STZ-induced diabetic rats, proliferative-stage lesions (e.g., intraretinal hemorrhage and preretinal neovascularization) only appear after six to nine months of sustained hyperglycemia [24]. Therefore, we were unable to fully evaluate the changes in senescence markers and the progression of neural dysfunction during the study period, leaving the long-term trajectory of DR unclear. Future studies with longer follow-up periods are warranted to elucidate the temporal dynamics of retinal senescence in diabetes. Third, we did not investigate senescence in other glial or neuronal cell types (e.g., Müller cells and amacrine cells) and their influence on retinal function. The pathogenesis of DR involves a wide range of cell types, including neurons, glia, and vascular components. For instance, Müller glial cells reportedly accumulate age-related transcripts more prominently than other retinal cell types [24]. Thus, the lack of evaluation of senescence in non-RGC populations represents a knowledge gap in the pathology of retinal aging. Our findings warrant cautious interpretation due to the absence of analyses incorporating senescence and functional changes in supporting and other neuronal cells. The methodological and model-related limitations of this study should be addressed in future investigations by incorporating electrophysiological parameters capable of directly assessing RGC function and by conducting long-term, comprehensive retinal analyses. These investigations will be essential to complement and expand the insights obtained in the present study.

## Conclusions

Cellular senescence is potentially involved in the pathogenesis of RGC degeneration caused by diabetes, as suggested by our findings. In STZ-induced diabetic mice, retinal dysfunction was associated with the upregulation of senescence markers and SASP, with a subset of RGCs in the GCL exhibiting senescence-associated changes. Additionally, oxidative stress and pharmacologically induced senescence directly impaired RGC structure in vitro. Thus, DR can be considered not only a vascular disorder but also a neurodegenerative disease. These findings provided insight into retinal aging and neuronal susceptibility in diabetes.
